# The Stroke Oxygen Study (SO_2_S) - a multi-center, study to assess whether routine oxygen treatment in the first 72 hours after a stroke improves long-term outcome: study protocol for a randomized controlled trial

**DOI:** 10.1186/1745-6215-15-99

**Published:** 2014-03-31

**Authors:** Christine Roffe, Tracy Nevatte, Peter Crome, Richard Gray, Julius Sim, Sarah Pountain, Linda Handy, Peter Handy

**Affiliations:** 1 Stroke Research, North Staffordshire Combined Healthcare NHS Trust, Holly Lodge, 62 Queens Road, Stoke on Trent, Staffordshire ST4 7LH, UK; 2 Stroke Research, Institute for Science and Technology in Medicine, Keele University, Keele ST5 5BG, UK; 3 Primary Care and Population Health, University College London Medical School (Royal Free Campus), Rowland Hill Street, London NW3 2PF, UK; 4 Clinical Trial Service Unit, University of Oxford, Roosevelt Drive, Oxford OX3 7LF, UK; 5 Health Services Research Unit, Keele University, Keele ST5 5BG, UK; 6 Stroke Research, Heart of England NHS Foundation Trust, Birmingham B9 5SS, UK; 7 Strokes R Us, High Lane, Stoke on Trent ST6 7DZ, UK

**Keywords:** Stroke, Oxygen supplementation, Oxygen, Hypoxia, Oximetry, Oxygen saturation

## Abstract

**Background:**

Mild hypoxia is common in stroke patients and may have significant adverse effects on the ischemic brain after stroke. The use of oxygen treatment is rapidly increasing in European stroke units but is not without side effects. It impedes early mobilization, could pose an infection risk, and may encourage the formation of toxic free radicals, leading to further damage to the ischemic brain. In the Stroke Oxygen Pilot Study (2 or 3 L/min for 72 hours) neurological recovery at one week was better in the oxygen group than in controls, and after correction for difference in baseline stroke severity and prognostic factors, there was a trend to better outcome with oxygen at six months. Oxygen was as effective in mild as in severe strokes.

Oxygen saturation is lower at night than during the day, and episodes of oxygen desaturation are common during sleep. Nocturnal oxygen supplementation is likely to reduce the burden of hypoxia without interfering with daytime mobilization and rehabilitation.

Before wider use of oxygen supplementation becomes established it is important to obtain better evidence on which patients benefit from such treatment.

**Methods:**

Participants will be randomized to one of three groups: the first will receive continuous oxygen for 72 hours (at a rate of 2 or 3 L/min depending on baseline oxygen saturation), the second group will receive nocturnal oxygen only (at a rate of 2 or 3 L/min depending on baseline oxygen saturation) and the third group will not receive any oxygen (control). A baseline assessment is performed at randomization and a one-week follow-up completed. Outcome data at three, six and twelve months will be obtained via a questionnaire sent to the patient by the trial center.

**Discussion:**

This study will provide evidence on the effectiveness of oxygen supplementation for the treatment of stroke and whether nocturnal oxygen is a potentially beneficial therapy regimen.

**Trial registration:**

This trial is registered with the ISRCTN register ID number ISRCTN52416964

## Background

### Why is there a need to investigate the effects of oxygen supplementation after stroke?

It is now well established that specialist care on stroke units is effective in preventing death and disability after stroke [1]. It remains unclear, however, which aspects of stroke care are crucial for improving outcome. It has been shown that patients on a stroke unit are more likely to receive oxygen than those on a non-specialized general ward [2]. Mild hypoxia is common in stroke patients and may have significant adverse effects on the ischemic brain after stroke [3]. Hypoxemia in the first few hours after hospital admission is associated with an increased risk of death [4]. While healthy adults with normal cerebral circulation can compensate for mild hypoxia with an increase in cerebral blood flow [5], this is not possible in the already ischemic brain after stroke [6-8]. The use of oxygen treatment is rapidly increasing in European stroke units. A questionnaire survey of UK stroke physicians showed that almost 50% of respondents would start oxygen supplementation after stroke at an oxygen saturation level of 95% or above [9], which is well within the normal physiological range [10]. In many UK Accident and Emergency departments, oxygen is given routinely to stroke patients irrespective of blood oxygen levels.

Oxygen treatment is not without side effects [11]. It impedes early mobilization and could pose an infection risk. There is evidence from animal models and *in vitro* studies that oxygen encourages the formation of toxic free radicals, leading to further damage to the ischemic brain [12-15], especially during reperfusion. Marked changes in adenosine triphosphate (ATP) and related energy metabolites develop quickly in response to acute ischemia and tissue hypoxia. These alterations are only partially reversed on reperfusion, despite improved oxygen delivery. Ischemia-induced decrease in the mitochondrial capacity for respiration results in reduced oxygen consumption and increased free radical generation during reperfusion [16]. Oxidative stress has also been implicated in the activation of cell-signaling pathways that lead to apoptosis and neuronal cell death [17,18]. While much research points towards adverse effects of hyperoxia in the ischaemic brain, there is also evidence to support the notion that therapy-induced eubaric hyperoxia may be neuroprotective [19,20]. Routine oxygen supplementation for acute myocardial infarction has been abandoned after a clinical trial showed no benefit, and potential harm [21]. A quasi-randomized study of oxygen supplementation for acute stroke by Ronning and Guldvog [22] has shown that routine oxygen treatment in unselected stroke patients does not reduce morbidity and mortality. Subgroup analyses within their study suggested that patients with severe strokes were more likely to benefit than those with mild strokes; however the study size was too small to define with certainty patients who are likely to derive benefit. A recent very small study [23] of high-flow oxygen treatment after acute stroke showed that cerebral blood volume and blood flow within ischemic regions improved with hyperoxia. Within 24 hours magnetic resonance imaging of the brain showed reperfusion in 50% of hyperoxia-treated patients versus 17% of controls (*p* = 0.06) but no long-term clinical benefit at three months [23]. In the recently completed Stroke Oxygen Pilot Study, the flow rate of oxygen was lower (2 or 3 L/min dependent on baseline oxygen saturation) and treatment was continued for longer (72 hours) [24,25]. Neurological recovery at one week was better in the oxygen group than in the controls. While there was no difference in outcome at six months on direct comparison, there was a trend for a better outcome with oxygen after correction for differences in baseline stroke severity and prognostic factors. Oxygen was as effective in mild as in severe strokes, in contrast to the earlier study by Ronning and Guldvog [22]. These results are promising, but need confirmation in a larger study.

Clinical guidelines on oxygen supplementation after stroke are not based on evidence from randomized clinical trials [26], differ from country to country, and change over time without obvious reason. The European Stroke Initiative (2008) suggests that routine oxygen supplementation to all stroke patients has not been shown to be effective, but that adequate oxygenation is important and that oxygenation can be improved by giving oxygen at a rate of >2 L/min (no target saturation or supporting evidence given) [27]. In 2003, the American Stroke Association Guideline recommended keeping the oxygen saturation level at or above 95% [28]. There was no change to the recommendations in the 2005 update of the guideline [29], but in 2007 the advice was revised to say that oxygen saturation should be maintained at or above 92% [30]. The latest UK National Clinical Guideline for the management of people with stroke (July 2008) [31] and the 2008 guidance from the National Institute for Clinical Excellence [32] state that supplemental oxygen should only be given to people who have had a stroke if the oxygen saturation falls below 95%. None of the recommendations are based on evidence from controlled clinical trials. Not surprisingly, there is uncertainty amongst physicians treating patients with stroke about which treatment approach to take and when to give oxygen, as shown by a recent survey of British Stroke Physicians [9].

For all the above reasons it is important to identify the groups of patients who benefit from oxygen, and the others who do not.

### What is the justification for the fixed dose oxygen regime suggested for this study?

A fixed dosage scheme has been chosen to keep the design of the study as simple as possible so that any recommendations resulting from the study outcome can be carried out in day-to-day clinical practice.

Ronning and Guldvog have shown that giving oxygen at a rate of 3 L/min to all stroke patients during the first 24 hours after hospital admission does not improve overall outcome [22]. They did not report baseline oxygen saturation or changes in saturation on treatment. It is therefore possible that some patients were undertreated and others achieved excessively high oxygen levels, leading to an increase in free radical generation in the ischemic penumbra [33]. There are no other data from clinical studies to inform recommendations for the dose of oxygen to give. The recently updated European Stroke Initiative suggests a dose of 2 to 4 L/min [34], and the American Stroke Association Guideline recommends keeping the oxygen saturation at or above 95% [28-30], but neither of these recommendations is based on evidence from controlled clinical trials. In the absence of data to the contrary it is reasonable to assume that treatment should restore oxygen saturation to the normal range.

Normal oxygen saturation for adults is 95.0 to 98.5% [35]; in healthy older individuals it is lower at 95% ± 2.5% [10]. Oxygen saturation in stroke patients who are normoxic at recruitment is about 1% lower than that of age-matched community controls [36]. A recently completed dose titration study for oxygen after acute stroke found that 2 L/min oxygen by nasal cannulae increases oxygen saturation by 2% and 3 L/min by 3% [37]. It was also found that oxygen masks were less likely to be tolerated than nasal cannulae, leading to poorer treatment compliance with the former. For this study it was therefore decided to give oxygen by nasal cannulae. A dosage regimen of 3 L/min for individuals with a baseline oxygen saturation of ≤93% and 2 L/min for individuals with a baseline saturation >93% is likely to prevent hypoxia without increasing oxygen saturation beyond the upper limit of the normal range.

### What are the advantages of giving routine oxygen supplementation at night only?

#### Patients are more likely to be hypoxic at night

The mean nocturnal oxygen saturation is about 1% lower than mean oxygen saturation when awake, in both stroke patients and controls [38]. A recent study in our unit has shown that a quarter of patients who are normoxic in the day have significant hypoxia during the night. About 60 to 70% of stroke patients suffer from sleep apnea early after the stroke [38-40].

#### The development of hypoxia is more likely to be missed at night

It is more difficult to observe patients in a darkened room and, unless there are reasons to suspect the patient is unwell, nurses will not wake the patient for routine observations. The development of hypoxia is therefore more likely to be missed at night.

#### Nocturnal hypoxemia is more likely to lead to brain tissue hypoxia at night

A recent study in normal volunteers has shown that hypoxemia leads to a compensatory increase in cerebral blood flow during wakefulness, but not during sleep, and is therefore more likely to result in brain tissue hypoxia at night [41].

#### Nocturnal oxygen supplementation does not interfere with the patient’s daytime mobility

Early mobilization is an important factor determining good outcome [2]. Patients who are attached to monitoring or oxygen supplementation equipment are less likely to be mobilized than patients who are not.

Giving routine oxygen only at night might prevent a significant number of otherwise undetected episodes of hypoxia, without interfering with the patient’s daytime rehabilitation.

## Methods/Design

### Study design

**Figure 1:**
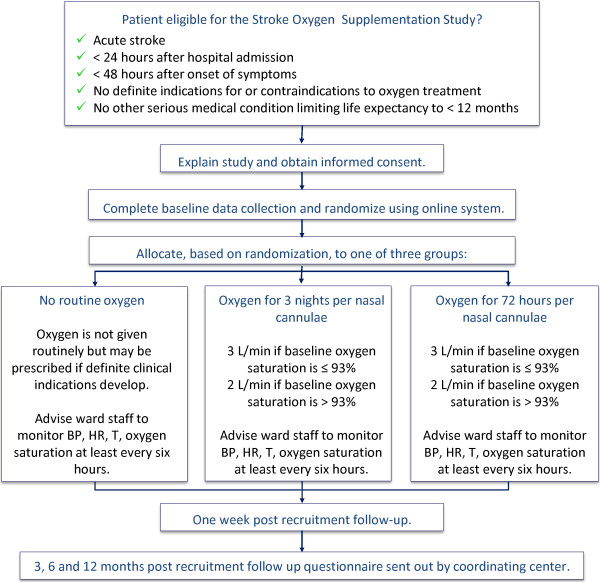
**The Stroke Oxygen Supplementation Study (SO**_**2**_**S) flowchart.** The SO_2_S randomizes stroke patients to one of three treatment groups, nocturnal oxygen for three nights, continuous oxygen for 72 hours or no oxygen. Patients are then followed-up at one-week, three, six and twelve months post randomization.

The design of the study is as follows: a multi-center, prospective, randomized, open, blinded-endpoint study of routine oxygen supplementation after acute stroke versus no routine oxygen treatment (Figure 1).

### Trial hypothesis

The primary hypothesis is that fixed dose oxygen treatment during the first three days after an acute stroke improves outcome after stroke.

The secondary hypothesis is that restricting oxygen supplementation to night time only is more effective than continuous supplementation.

### Recruitment

Patients will be recruited from multiple (>30) centers throughout the UK and worldwide. The first study center to be enrolled will be the University Hospital of North Staffordshire. Centers will be eligible for participation in the study if they admit patients with acute stroke, are able to provide oxygen treatment and monitor oxygen saturation, and if there is a local researcher who will act as the principal investigator for the locality.

### Inclusion criteria

All adult patients with an acute stroke will be eligible to be considered for study participation. There are no definite guidelines for oxygen treatment after acute stroke, and there is uncertainty among stroke physicians about who should be given oxygen and for how long. The eligibility criteria for inclusion in the trial reflect this uncertainty, and allow for randomization of all acute stroke patients who do not have definite indications for or definite contraindications to oxygen treatment.

Hence, adult patients will be eligible for trial inclusion if they were admitted with symptoms of an acute stroke within the preceding 24 hours, and in the doctor’s opinion there is no clear indication for, and no clear contraindication to oxygen treatment.

The diagnosis of stroke will be made by history and clinical examination and is at the discretion of the admitting doctor. It will based on the World Health Organization (WHO) criteria (rapidly developing clinical signs of focal or global disturbance of cerebral function, with symptoms lasting 24 hours or longer, or leading to death, with no apparent cause other than of vascular origin) [42]. Within the first 24 hours of symptom onset a definite distinction between a stroke and a transient ischemic attack cannot be made. However, most patients who still have persistent symptoms after one hour will be confirmed to be having a stroke. Since waiting for 24 hours for confirmation would unnecessarily delay treatment, we omitted the time element from the definition of stroke for the purposes of trial inclusion.

### Exclusion criteria

Patients will be excluded from the trial if the responsible doctor considers the patient to have definite indications for, or contraindications to, oxygen treatment at a rate of 2 to 3 L/min. The decision will be left to the responsible clinician. This exclusion criterion has been chosen to ensure that all patients are treated according to best medical practice.

Potential indications for oxygen treatment could be: oxygen saturation on air <90%, hypoxia associated with acute left ventricular failure, severe pneumonia, pulmonary embolus, and chronic respiratory failure treated with long term oxygen at home.

Potential contraindications to fixed dose oxygen treatment could be type two respiratory failure and very severe hypoxia.

Patients will also be excluded if the stroke is not the main clinical problem, or if they have another serious life-threatening illness likely to lead to death within the subsequent few months. This group of patients is excluded because it is unlikely that they are going to derive any benefit from the trial treatment.

### Intervention

Patients will be randomized to one of three treatment groups:

**Treatment group 1:** no routine oxygen supplementation during the first 72 hours after randomization.

**Treatment group 2:** oxygen per nasal cannulae overnight (9 pm to 7 am) at a flow rate of 3 L/min (if baseline oxygen saturation is 93% or below) or at a flow rate of 2 L/min (if baseline oxygen saturation is greater than 93%) during the first three nights after randomization.

**Treatment group 3:** oxygen per nasal cannulae continuously (day and night) at a flow rate of 3 L/min (if baseline oxygen saturation is 93% or below) or a flow rate of 2 L/min (if baseline oxygen saturation is greater than 93%) during the first 72 hours after randomization.

All patients will have regular observations of vital signs (blood pressure, heart rate, temperature and oxygen saturation) every six hours or less, as per the local protocol of the stroke unit. Treatment of any abnormal findings will be independent of trial allocation. Patients who require oxygen, or changes in the dose of oxygen, for clinical reasons at any time of the trial will be given the concentration of oxygen they require.

### Blinding

This study will be open, since placebo treatment (room air) would have similar side effects as the active treatment (such as infection and immobilization), and would thus bias the data in favor of the treatment group. The main outcomes will be ascertained at three months by central follow-up, ensuring that the assessor is blind to the intervention. When the patients complete the questionnaire they may have some recollection of being treated with oxygen or not. Patients will be asked to state on the questionnaire if they remember or can guess which treatment group they were in. This will then be compared with the actual allocation to quantify potential bias.

### Initial assessment

The initial assessment will be done by the researcher randomizing the patient, or entered online for patients randomized via the web, or sent to the trial center by fax for patients randomized via the telephone. It will include: baseline demographics, date and time of event, the Glasgow Coma Scale [43], predictors of outcome [44] and the National Institutes of Health Stroke Scale (NIHSS) [45,46].

### Follow-up assessments

#### Week one

The one-week assessment will confirm the diagnosis, and will document deaths and neurological status (NIHSS), compliance with the intervention, and complications. It will be performed by a member of the local research team trained in the assessment tools seven days (± one day to allow for weekends and holidays) after enrolment. In the case of patients who are discharged before the end of one week, or who cannot be followed at seven days, the patient will be assessed at discharge and if appropriate the follow-up completed at this time point. Wherever possible we will strive to assess the patient at day seven after randomization in hospital or, if discharged, in clinic or in their place of residence as the patient specifies. If the one-week assessment is not possible then the discharge assessment is acceptable as the one-week outcome. Data will be entered online or sent to the trial center via fax.

#### Three months, six months and twelve months

The main follow-up will be performed centrally at three months, a standard procedure for most acute stroke trials. Assessments will be based on a questionnaire sent to the patient’s preferred follow-up address by the central team, after checking with the GP that the patient is still alive, unless the patient has specified a different preference at the one-week assessment. Central follow-up will ensure blinding of the assessors to the intervention. For non-responders the address will be checked via the GP and the local researcher and then resent. If there is no response, patients will be contacted by phone to see if they would prefer a personal follow-up or are happy to reply to questions via the telephone. Missing or inconsistent data will be cross-checked with the medical notes, with the GP, or by personal contact with the patient. If patients are not contactable using these methods, we will determine if they have died and, if so, what the cause of death was by requesting information from the Office of National Statistics or the NHS Strategic Tracing Service. The latter will also be contacted if the patient is no longer resident at the address given and the GP has not seen the patient recently and does not know the patient’s new address and new GP.

The follow-up questionnaire will contain the modified Rankin score (mRS) [47], Barthel Index of Activities of Daily Living Score (ADL) [48], Nottingham Extended Activities of Daily Living (NEADL) index [49], EQ-5D^TM^[50-52], and questions regarding memory, sleep, speech and discharge status.

#### Outcome measures

The primary outcome is measured using the mRS score at three months [53]. Secondary outcomes are measured at one week using: the number of patients with neurological improvement (≥4 point decrease in the NIHSS) [54], the number of deaths, the highest oxygen saturation during the first 72 hours, and the lowest oxygen saturation during the first 72 hours. Further secondary outcomes are measured at three months using: the mortality rate, the percentage of patients living at home, the Barthel ADL score, the EuroQol score and the Nottingham Extended Activities of Daily Living scale (NEADL). Further explanatory analyses include: antibiotic and sedative use during week one, the highest heart rate during the intervention >100, the highest systolic blood pressure during the intervention >200 mm Hg, the highest diastolic blood pressure during the intervention >100 mm Hg, oxygen saturation during the intervention, the percentage of patients describing their memory as ‘as good as before the stroke’, the percentage of patients describing their sleep as ‘as good as before the stroke’, the percentage of patients without significant speech problems, and the change in outcomes over time (three month assessments repeated at six and twelve months).

Those outcomes concerning memory, sleep and speech have been highlighted as important on the public consultation [55], but are not part of standard assessment scales for stroke outcome.

All study measures will be performed by research staff appropriately trained in the use of the assessment tools.

### Data management and evaluation

#### Randomization

Patients will be randomized using minimized randomization stratified by well validated prognostic factors (age, sex, living alone, normal verbal component of the GCS, ability to lift both arms and ability to walk) [56,57], routine oxygen treatment during ambulance transfer, and baseline oxygen saturation, via a web-based randomization system. Randomization will not be stratified by the study center as this may result in unacceptably high rates of allocation prediction and selection bias [58]. However, retrospective analysis by center will be performed to investigate any heterogeneity of treatment effect by center.

#### Data management

The local investigators, their research assistants, and data monitors will have access to the patient records. Personalized data (address, telephone number, email and fax) will be kept in the trial office, as electronic copies within each of the centers, and at the coordinating center to allow patients to be contacted for the three-, six- and twelve-month follow-up and to allow data checks and validation.

For all other purposes patient identifiable data will be converted to an alphanumeric code, using a specific code number for each patient. The principal investigators, members of the trial steering group, the trial managers, data managers, data monitors, the data analysts/programmers, and the trial statisticians will have access to the anonymized data.

Data will be stored on password protected office computers and on Zip discs, flash drives or CD-ROMs. At least three back-up copies will be made of all data. These will be kept in locked cupboards in separate buildings.

Data will be transmitted via fax, email or the web from each local center to the trial coordinator and data queries will be transmitted via the same route from the trial coordinator to local centers. Anonymized data may be made available to other researchers for meta-analysis and publication in media such as the Cochrane Database.

#### Statistical analysis

The analysis will be by intention to treat. The primary outcome is the mRS score, which has an ordinal range of 0 (best outcome) to 5 (worst outcome). This will be measured at three months (or at the last rating). Deaths will be allocated an arbitrary score of 6 [58]. A later primary outcome assessment at six months was considered, but rejected because there is a risk of diluting treatment effects by newly occurring health problems unrelated to the trial intervention [59].

The trial tests two research hypotheses: 1. oxygen supplementation results in better (lower) mRS scores at three months than no oxygen supplementation; 2. Oxygen administered at night results in better mRS scores than oxygen given over a 24 hour period.

It cannot be assumed that any benefits from oxygen will be dose dependent. Oxygen supplementation throughout the 24-hour period may expose a significant number of patients to oxygen concentrations higher than normal. Being attached to oxygen may also limit mobility and hinder early mobilization. Oxygen given at night only will provide supplementation at a time when oxygen saturation is lowest in stroke patients, and will not interfere with rehabilitation. A prior hypothesis is that oxygen at night will have all the potential advantages without the disadvantages associated with daytime oxygen use.

The mRS will be compared between two groups using ordinal logistic regression [60]. Both an unadjusted and an adjusted analysis will be performed. The latter will incorporate the following covariates: age, sex, baseline NIHSS score, and the ‘six simple variables’ (SSV) prognostic index for independence at six months. The SSV score is based on the following variables: age, living alone before the stroke, independent pre-stroke, normal verbal response to questions, able to lift the affected arm against gravity, and able to walk unaided [56,57]. Planned subgroup analyses for the primary outcome are: baseline SSV prognostic index (<0.2, 0.2 to 0.35, 0.36 to 0.7, >0.7), NIHSS score at baseline (<5, ≥5), baseline oxygen saturation (<95, ≥95), treatment with oxygen prior to randomization (yes or no), time since onset of stroke in hours (<4, 4 to 6, 7 to 12, 13 to 24, >24), type of stroke (hemorrhage, infarction). Mortality will be assessed using survival analysis, and other secondary outcomes will be analyzed using appropriate statistical methods. All tests will be two-tailed and 95% confidence intervals will be calculated for all estimates of effect.

#### Study size

Many acute stroke studies have been underpowered because the expected treatment effect was unrealistically large [61]. While thrombolysis within three hours of acute stroke has been shown to lead to moderate clinical benefits (0.5 points on the mRS) [58], neuroprotectant treatments may well achieve lesser (for example, 0.2 point on the mRS) treatment effects [62]. As stroke is such a common condition and oxygen supplementation is inexpensive and universally available, even relatively small differences in outcome could have a major impact on the burden of the disease. For example, treating five patients with an average improvement of 0.2 mRS would improve one patient by one mRS category (for example from moderate disability to slight disability). However though important, small differences do require very large trials in order to show effectiveness.

The sample size calculation is based on an odds ratio of 0.83 for a more adverse outcome (higher mRS score) for the oxygen and control groups, observed in the first 200 patients in the Stroke Oxygen Pilot Study (ISRCTN12362720) [24,25] (C Roffe, P Jones, S Sills, personal communication). The sample size allows for a 5% drop-out rate for such things as retrospective exclusions for a change of diagnosis (numbers based on the Stroke Oxygen Pilot study) plus a 5% rate of missing outcome data (this overall 10% loss to follow-up gives a safe margin; target would be less than 3%).

A sample size of 6,000 patients will therefore provide 95% power to detect the specified odds ratio between oxygen (continuous and night only groups combined) and no oxygen at *p* ≤ 0.05 (two-tailed), and 87% power at *p* ≤ 0.05 (two-tailed) to detect the same effect between continuous oxygen and oxygen at night only. Adjustment for a maximum 10% loss to follow-up gives a target sample size of 6,669.

#### Increase of sample size in October 2012

A sample size of 8,000 patients will be used, as increasing the recruitment target would give greater power to detect an interaction between subgroups (defined by severity) and the effect of oxygen versus control. The magnitude of the increase in power can be estimated by considering the increase in power to detect the pre-specified odds ratio of 0.83 in the subgroup of ‘moderate through to very severe’ patients (those with an NIHSS score greater than or equal to 10, who are more likely to benefit from the treatment). Using a revised loss to follow-up rate of 5%, power to detect the specified odds ratio between oxygen and control in this subgroup would rise from 43% to 50%. For the whole sample, power to detect this effect between oxygen treatment and no oxygen treatment will rise from 95% to approximately 98% and that for the comparison of continuous oxygen and oxygen at night will rise from 87% to approximately 94%.

### Ethical requirements

#### Approval of the study by the research ethics committee

Multicenter ethical approval was granted for version 2 of the protocol by the North Staffordshire Research Ethics Committee on 25 June 2008 (COREC 06/Q2604/109).

#### Good clinical practice

The study will be performed in accordance with the principles stated in the Declaration of Helsinki [63]. Study procedures will be guided by the standards outlined in the MRC Guidelines for Good Clinical Practice in Clinical Trials [64].

#### Patient information and consent

Consent will be obtained according to the requirements of the Multicentre Research Ethics Committee and the Local Research Ethics Committee before the start of recruitment.

The patient, and where appropriate the next of kin, will be given full and adequate oral and written information about the nature and purpose of the study, possible risks and benefits. A copy of the patient information sheet and the signed consent/assent will be given to the patient. Patients will be informed that they are free to discontinue participation in the study at any time.

Fully informed consent will be sought from all competent subjects. In patients who are conscious, but not fully competent to understand the information to make a reasoned decision, we will provide a simple explanation of the trial and seek the patient’s agreement, and also seek assent from the next of kin or from an independent physician. If an incompetent individual has been included in the trial without giving fully informed consent we will strive to obtain fully informed consent as soon as the patient is able to do so. This will be documented on the one-week follow-up form.

The reason for including patients unable to give fully informed consent is that roughly one third of stroke patients will have problems with speech and with the understanding of spoken and written material as a consequence of their stroke. It is important to include these patients in the study since they are just as likely to benefit from the treatment as patients who are able to communicate.

It is further important to include as wide a spectrum of stroke patients as possible, in particular patients with severe strokes. Patients with severe strokes may be more likely to develop hypoxia, and may therefore be more likely to benefit from oxygen treatment than patients with mild strokes. However, patients with severe strokes are more likely to be confused, drowsy or dysphasic, and thus unable to give informed consent. Exclusion of subjects unable to give informed consent is thus likely to bias trial outcome. Furthermore, since the group of patients who are unable to consent has different clinical characteristics from patients who can give consent, the results of the study may not be applicable to patients with similar clinical presentations to the excluded patients.

The information sheets to be given to patients, relatives, or the independent clinician have been reviewed and edited by service users from Strokes R Us (Stoke-on-Trent, UK) and Different Strokes (Coventry, UK).

#### Monitoring of suspected unexpected serious adverse reactions

All suspected unexpected serious adverse reactions (SUSARs) that are believed to be due to the trial treatment will be reported as soon as possible within one working day of the clinician becoming aware of the event by phoning the study helpline or by emailing christine.roffe@northstaffs.nhs.uk. A SUSAR report form will be completed as fully as possible and sent, via fax, to the Chief Investigator and the sponsor (North Staffordshire Combined Healthcare Trust Research and Development). On this form the patient will be identified by a unique identifying number consisting of the trial identification number (ISRCTN) followed by the number the patient was allocated at randomization. The SUSAR form will be filed in the trial master file and in the case record form. A SUSAR follow-up form will be completed as soon as possible within five days of the event and submitted via fax to the coordinating center and sponsor. This will also be filed in the trial master file and the case record form. Unless the event has resolved or a decision has been taken that no further follow-up is required, further follow-up forms will be completed, faxed and filed as outlined above until the event has resolved. Relevant details of the SUSAR and its follow-up will also be recorded in the patient’s medical notes. The sponsor will inform the licensing authority, the competent authorities of any member state in which the trial is being conducted, and the relevant Research Ethics Committee (West Midlands – Staffordshire Research Ethics Committee, Barlow House, 3rd Floor, 4 Minshull Street, Manchester, M1 3DZ, Fax 01785 254 640) of the SUSAR as soon as possible, and no later than seven days after first becoming aware of the event. The sponsor will provide details of follow-up reports and resolution. The sponsor will also inform all the principal investigators of the trial of the SUSAR. At the end of each year from the start of the trial the sponsor will provide the licensing authority (Medicines and Healthcare products Regulatory Authority, UK) with a list of all SUSARs relating to the trial during that year and any other relevant new information relating to the investigational product which may affect the conduct of this trial.

#### Data protection

Data will be stored and analyzed in accordance with national data legislation. Personalized data (address, telephone number, email and fax) will be kept in the trial office and as electronic copies within each of the centers and at the coordinating center to allow patients to be contacted for follow-up, and to allow data checks and validation. For all other purposes patient identifiable data will be converted to an alphanumeric code, using a specific code number for each patient.

### Trial administration

#### Sponsor

North Staffordshire Combined Healthcare NHS Trust, Trust Headquarters, Bellringer Road, Trentham, ST4 8HH.

#### Person authorized by the sponsor to act on behalf of the sponsor

R&D Director, North Staffordshire Combined Healthcare NHS Trust, Trust Headquarters, Bellringer Road, Trentham, ST4 8HH. Tel: 01782 441651.

#### Chief investigator

Professor C Roffe, North Staffordshire Combined Healthcare NHS Trust, Holly Lodge, 62 Queens Road, Hartshill, Stoke-on-Trent ST4 7LH. Tel 0300 123 0891 Fax 0300 123 0894, email: christine.roffe@northstaffs.nhs.uk.

#### Trial management committee

The trial management committee (TMC) is responsible for the overall design and conduct of the study, analysis of the data, reporting and dissemination of results. It will act on advice of the trial steering committee, the data safety and management committee, the advisory groups and the international advisory committee.

Membership: Professor C Roffe (chair, stroke physician, clinical lead of the West Midlands Local Stroke Research Network); Professor P Crome (geriatrician, clinical trialist and pharmacologist), Primary Care & Population Health, University College London; Professor R Gray (expertise in large clinical trials), University of Oxford; Professor J Sim (statistician), Keele University; Professor P Jones (statistician), Keele University; Mr Peter and Mrs Linda Handy (patient representatives), Strokes R Us, Stoke-on-Trent.

#### Trial steering committee

The trial steering committee (TSC) will oversee the study. Professor M Dennis (stroke physician, clinical trialist), University of Edinburgh will act as independent chairman. Other members are: Professor L Kalra (stroke physician, clinical trialist), King’s College, London; Professor S Maslin-Prothero (nursing, policy and practice in the NHS); J Daniels (clinical trialist), Birmingham Clinical Trials Unit; Mrs P Bell (patient representative, dysphasia support); Professor R Lindley (international advisor, stroke physician, clinical trialist); and members of the TMC.

#### Data monitoring and safety committee

The remit of the Data Monitoring and Safety Committee (DMSC) will be to ensure that patients are not exposed to unnecessary risks, by performing interim safety analyses, and to maintain patient safety. If oxygen treatment really provides substantial benefit or harm with respect to the primary endpoints, then this may become apparent before the target recruitment has been reached. Alternatively, new evidence might emerge from other sources that oxygen is definitely effective, ineffective, or adverse. To protect against this, during the period of recruitment to the study, interim analyses of major endpoints will be supplied, in strict confidence, to an independent Data Monitoring and Safety Committee along with updates on results of other related studies, and any other analyses that the DMSC may request. The DMSC will advise the chair of the TSC if, in their view, the randomized comparisons in the trial have provided both (a) ‘proof beyond reasonable doubt’ that for all, or for some, types of patient one particular treatment is definitely indicated or definitely contraindicated in terms of a net difference in the major endpoints; and (b) evidence that might reasonably be expected to influence the patient management of many clinicians who are already aware of the other main trial results. Appropriate criteria of proof beyond reasonable doubt cannot be specified precisely, but a difference of at least three standard deviations in an interim analysis of a major endpoint may be needed to justify halting, or modifying, the study prematurely. If this criterion were to be adopted, the exact number of interim analyses is of little importance, so no fixed schedule is proposed. The TSC can then decide whether to close or modify any part of the trial. Unless this happens, however, the TMC, the TSC, the investigators and all of the central administrative staff (except the statisticians who supply the confidential analyses) will remain unaware of the interim results.

The DMSC is chaired by Professor S Jackson (pharmacology, prescribing, clinical trials), King’s College London. Other members will be Professor T Robinson (stroke physician, clinical lead of the Trent Stroke Local Research Network), University of Leicester, and Dr M Lewis (statistician), Keele University.

## Trial status

The Stroke Oxygen Study has completed recruitment and is in follow-up.

## Abbreviations

ADL: Activities of Daily Living; ATP: Adenosine triphosphate; DMSC: Data monitoring and safety committee; EQ-5DTM: EuroQoL (quality of life); GCS: Glasgow Coma Scale; MRC: Medical Research Council; NEADL: Nottingham Extended Activities of Daily Living; NIHSS: National Institutes of Health Stroke Scale; SO2S: The Stroke Oxygen Study; SSV: six simple variables; SUSAR: Suspected unexpected serious adverse reaction; TMC: Trial Management Committee; TSC: Trial Steering Committee; WHO: World Health Organization.

## Competing interests

The authors declare that they have no competing interests.

## Authors’ contributions

CR: conception and design of work, manuscript writing and final approval of manuscript. TN: design of work, manuscript writing and final approval of manuscript. PC: conception and design of work, critical revision and final approval of manuscript. RG: design of work, critical revision and final approval of manuscript. JS: design of work, critical revision and final approval of manuscript. SP: design of work, critical revision and final approval of manuscript. LH: design of work, critical revision and final approval of manuscript. PH: design of work, critical revision and final approval of manuscript. All authors read and approved the final manuscript.
